# High-precision and cost-efficient sequencing for real-time COVID-19 surveillance

**DOI:** 10.1038/s41598-021-93145-4

**Published:** 2021-07-01

**Authors:** Sung Yong Park, Gina Faraci, Pamela M. Ward, Jane F. Emerson, Ha Youn Lee

**Affiliations:** 1 Department of Molecular Microbiology and Immunology, Keck School of Medicine, University of Southern California, Los Angeles, CA USA; 2 Department of Clinical Pathology, Keck School of Medicine, University of Southern California, Los Angeles, CA USA

**Keywords:** Viral infection, Molecular medicine

## Abstract

COVID-19 global cases have climbed to more than 33 million, with over a million total deaths, as of September, 2020. Real-time massive SARS-CoV-2 whole genome sequencing is key to tracking chains of transmission and estimating the origin of disease outbreaks. Yet no methods have simultaneously achieved high precision, simple workflow, and low cost. We developed a high-precision, cost-efficient SARS-CoV-2 whole genome sequencing platform for COVID-19 genomic surveillance, CorvGenSurv (Coronavirus Genomic Surveillance). CorvGenSurv directly amplified viral RNA from COVID-19 patients’ Nasopharyngeal/Oropharyngeal (NP/OP) swab specimens and sequenced the SARS-CoV-2 whole genome in three segments by long-read, high-throughput sequencing. Sequencing of the whole genome in three segments significantly reduced sequencing data waste, thereby preventing dropouts in genome coverage. We validated the precision of our pipeline by both control genomic RNA sequencing and Sanger sequencing. We produced near full-length whole genome sequences from individuals who were COVID-19 test positive during April to June 2020 in Los Angeles County, California, USA. These sequences were highly diverse in the G clade with nine novel amino acid mutations including NSP12-M755I and ORF8-V117F. With its readily adaptable design, CorvGenSurv grants wide access to genomic surveillance, permitting immediate public health response to sudden threats.

## Introduction

Pathogen whole genome sequencing informs evidence-based public health decisions by providing crucial data for disease transmission, new outbreak detection, and vaccine candidate selection^1,2^, as demonstrated by HIV^3^, tuberculous^4^, Ebola^5,6^ and Zika^7^ outbreaks. In the immediate response to COVID-19, several studies have demonstrated that genomic surveillance outcomes were not only comparable to epidemiological contact tracing data^8–10^ but also capable of tracing previously unknown linked transmissions^11^. Genomic investigation informed the public health decisions to prevent further spread of SARS-CoV-2, including travel restrictions and stay-at-home orders in response to the identification of travel-related clusters and local clusters^10^. Rapid SARS-CoV-2 whole genome sequencing is therefore an essential public health measure.

SARS-CoV-2 whole genome sequencing also has important utility in the surveillance of amino acid mutations that may result in changes to viral protein physical structures^12,13^. Such changes in structure can potentially alter virus transmissibility^14^, disease severity^15^, or reduce vaccine efficacy^16,17^. Over 90 vaccines are currently in development^18^ and spike protein based vaccine candidates have induced both neutralizing antibody and T-cell responses against SARS-CoV-2^19^. However, diverse mutations in viral proteins, as observed in GISAID^20,21^ and Nextstrain^22^, may lower COVID-19 vaccine effectiveness^16,17^, as observed in influenza vaccines^23,24^. Similarly, mutations in therapeutic drug target regions such as RNA-dependent RNA polymerase (RdRP) can potentially lead to treatment failure^25^. Therefore, it is important to be able to surveil virus mutations in real-time to ensure the efficacy of prophylactic vaccines and therapeutic drugs.

The greatest barriers to the deployment of massive SARS-CoV-2 whole genome sequencing for routine use are the complexity and expense of the workflows. Current SARS-CoV-2 whole genome sequencing methods, including the widely-used Artic network protocol, generally rely on short-read amplifications and sequencing^8,10–12,26–31^. However, a high number of short-read amplifications increases the risk of genome coverage dropouts^8^. Minimizing this risk often requires deep sequencing coverage, resulting in high-sequencing cost. To date, no SARS-CoV-2 whole genome sequencing method has concurrently accomplished high-resolution, simple workflow, and low-cost.

Herein, we introduce CorvGenSurv (Coronavirus Genomic Surveillance), an accurate and cost-efficient SARS-CoV-2 whole genome sequencing platform for COVID-19 genomic surveillance. We directly amplify viral RNA and sequence it in three segments using long-read, high-throughput sequencing. Sequencing of the SARS-CoV-2 whole genome in three segments prevents dropouts in genome coverage by minimizing ambiguous reads and eliminating assembly errors. CorvGenSurv is a streamlined, high-resolution, and cost-effective pipeline that facilitates the deployment of real-time SARS-CoV-2 whole genome sequencing for public health.

## Results

### Workflow of CorvGenSurv

**Figure 1:**
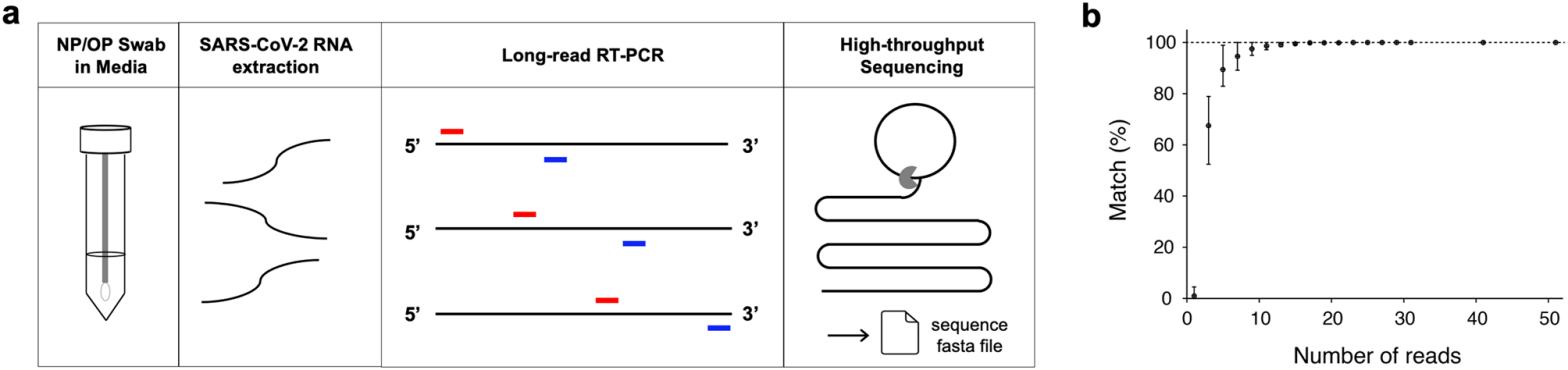
CorvGenSurv’s workflow and precision. **(a)** Remnant NP/OP specimens from COVID-19 diagnostic testing were subject to SARS-CoV-2 RNA extraction. Viral RNA was amplified via three overlapping RT-PCRs (~ 10,000 base long each) and pooled SARS-CoV-2 amplicons of indexed COVID-19 specimens were then sequenced by long-read high-throughput single-molecule sequencing. The output fasta file was de-multiplexed and processed to produce the consensus sequence of each segment. Each COVID-19 specimen’s three overlapping segments were assembled into a SARS-CoV-2 whole genome sequence. (**b)** CorvGenSurv’s precision was tested by comparing the consensus sequence from a given number of reads with the USA-WA1/2020 control strain (GenBank: MN985325.1). When a consensus sequence was built from three reads, only 67% [52.4–78.9%] of the 1000 bootstrap runs’ resulting consensus sequences were consistent with the correct sequence. When the number of the reads was greater or equal to 31, all 1000 bootstrap runs resulted in the correct sequence.

We extracted SARS-CoV-2 RNA from remnant COVID-19 positive NP/OP swab specimens in universal viral transport media. Rather than converting viral RNA to cDNA, we instead directly amplified viral RNA via long-read reverse transcriptase-polymerase chain reaction (RT-PCR). Three overlapping RT-PCR amplicons were produced for each specimen. Each specimen’s three overlapping RT-PCR amplicons were then indexed, and multiplexed specimens sequenced by long-read high-throughput sequencing^32,33^ (Fig. 1a). After de-multiplexing, we built the consensus sequence of each of the three segments, correcting sequencing errors. These overlapping consensus sequences were then assembled into a near full-length SARS-CoV-2 whole genome sequence for each COVID-19 case.

### Precision and accuracy of CorvGenSurv

We measured the accuracy of CorvGenSurv by sequencing the genomic RNA of the USA-WA1/2020 control strain (GenBank: MN985325.1) provided by BEI Resources (NR-52285). The assembled near full-length SARS-CoV-2 sequence obtained by CorvGenSurv matched 100% to the sequence of this control strain. This accuracy was achieved by our error correction step during consensus sequence building. To assess how many reads are required to produce an accurate consensus sequence, we performed a bootstrap test, randomly sampling a given number of reads and building the consensus sequence of those reads. As shown in Fig. 1b, when three reads were sampled, only 67% [52.4–78.9%] of the 1000 bootstrap runs’ resulting consensus sequences were consistent with the reference sequence of USA-WA1/2020. When we generated a consensus sequence from 31 or more reads, the consensus sequence of all 1000 bootstrap runs was identical to the control sequence. This 31-fold sequencing depth ensured the production of a SARS-CoV-2 whole genome sequence that perfectly matched the control sequence.

We also spot-checked the precision of CorvGenSurv by Sanger sequencing, the current gold standard for precision checking^34^. USA/CA-LAC-USC1 in Table 1 was selected, and a near full-length SARS-CoV-2 sequence was obtained by assembling a total of 49 overlapping segments that were sequenced by Sanger sequencing. The resulting Sanger sequence matched 100% to the corresponding USA/CA-LAC-USC1 sequence obtained by CorvGenSurv.

**Table 1:** Amino acid mutations, clade and lineage of 25 whole genome sequences from COVID-19 remnant NP/OP specimens.

Specimen ID	Collection date	C_t_-1	C_t_-2	Amino acid mutations	GISAID clade (Pango lineage)
USA/CA-LAC-USC1	4/13/20	18.58	18.72	NSP12-P323L**, NSP12-M755I,** S-D614G, N-R203K, N-G204R	GR (B.1.1)
USA/CA-LAC-USC2	4/13/20	24.84	25.44	NSP3-G1011X, **NSP3-F1659L**, NSP4-S312N, NSP12-P323L, S-D614G, N-R203K, N-G204R	GR (B.1.1)
USA/CA-LAC-USC3	4/14/20	24.18	24.96	NSP2-T85I, NSP12-P323L, NSP14-G44D, NSP16-S33R, S-D614G, S-D1184N, ORF3a-Q57H	GH (B.1)
USA/CA-LAC-USC4	5/5/20	20.06	20.46	NSP2-T85I, NSP12-P323L, S-D614G, ORF3a-Q57H	GH (B.1)
USA/CA-LAC-USC5	5/6/20	24.94	25.09	NSP3-G638S, NSP5-K90R, NSP6-Q208H, NSP12-P323L, NSP14-V287F, **ORF8-V117F**, S-F565L, S-D614G, N-R203K, N-G204R	GR (B.1.1.61)
USA/CA-LAC-USC6	5/9/20	28.25	28.48	NSP12-T85I, NSP12-P323L, NSP16-A188S, S-D614G, N-R203K, N-G204R	GR (B.1.1)
USA/CA-LAC-USC7	5/9/20	23.05	23.19	NSP3-D1214N, NSP12-P323L, S-D614G, N-R203K, N-G204R	GR (B.1.1)
USA/CA-LAC-USC8	5/18/20	17.34	17.10	NSP2-T85I, NSP12-P323L, S-D614G, S-P812L, ORF3a-Q57H, M-A68S, N-G34W	GH (B.1)
USA/CA-LAC-USC9	6/1/20	20.26	20.46	NSP2-K110N, NSP2-P191S, NSP12-P323L, S-D614G, ORF7a-H73R, N-S194L	G (B.1.397)
USA/CA-LAC-USC10	6/2/20	23.34	23.69	NSP2-K110N, NSP2-P191S, NSP12-P323L, S-D614G, N-S194L	G (B.1.397)
USA/CA-LAC-USC11	6/4/20	19.21	19.04	NSP12-P323L, S-D614G, ORF8-I47F, N-R203K, N-G204R	GR (B.1.1.172)
USA/CA-LAC-USC12	6/5/20	16.79	16.42	NSP3-Q203H, NSP12-P323L, NSP15-E223G, S-D614G, **N-P122H**, N-R203K, N-G204R	GR (B.1.1.228)
USA/CA-LAC-USC13	6/6/20	23.60	24.01	NSP2-T85I, NSP2-A361V, NSP8-L35F, NSP12-P323L, NSP13-K460R, NSP16-M17I, S-D614G, S-K1191N, ORF3a-Q57H, ORF3a-T175I	GH (B.1.166)
USA/CA-LAC-USC14	6/8/20	15.61	15.51	**NSP3-P192H**, NSP3-T1288I, NSP12-P323L, NSP13-P238S, S-D614G, N-Q9H, N-S194L	G (B.1)
USA/CA-LAC-USC15	6/9/20	18.59	18.64	**NSP3-I1672S**, NSP8-S177L, NSP12-P323L, S-D614G, S-P807R	G (B.1)
USA/CA-LAC-USC16	6/9/20	26.52	27.18	NSP12-P323L, S-T286I, S-A522V, S-D614G, ORF3a-Q57H	GH (B.1.110)
USA/CA-LAC-USC17	6/9/20	29.39	30.39	NSP1-V116M, NSP2-T85I, NSP3-A231V, NSP5-L89F, NSP12-P323L, NSP16-V294F, S-D614G, ORF3a-Q57H, ORF8-S24L	GH (B.1.595)
USA/CA-LAC-USC18	6/9/20	17.07	16.83	NSP2-K110N, NSP2-P191S, NSP12-P323L, NSP16-P236S, S-Y144X, S-D614G, N-S194L	G (B.1.397)
USA/CA-LAC-USC19	6/10/20	26.27	26.37	NSP12-P323L, S-D614G, ORF7a-P34S, N-R203K, N-G204R	GR (B.1.1)
USA/CA-LAC-USC20	6/10/20	27.65	28.42	NSP12-P323L, S-G142C, S-R214C, S-D614G, ORF3a-P159S, N-R203K, N-G204R, N-Q229H	GR (B.1.1.132)
USA/CA-LAC-USC21	6/11/20	24.27	24.82	NSP2-T85I, NSP3-P108L, NSP12-P323L, S-R21I, S-Y28H, S-D614G, ORF3a-Q57H, ORF8-S24L, N-G34W	GH (B.1.336)
USA/CA-LAC-USC22	6/11/20	24.48	25.19	NSP2-T85I, **NSP3-S702F**, NSP3-T1830I, **NSP4-Q77H**, NSP12-P323L, S-D614G, ORF3a-Q57H	GH (B.1)
USA/CA-LAC-USC23	6/22/20	29.29	29.45	NSP2-T85I, NSP12-T293I, NSP12-P323L, S-V308L, S-D614G, ORF3a-Q57H, N-S183Y	GH (B.1.369)
USA/CA-LAC-USC24	6/22/20	28.15	28.19	NSP12-P323L, NSP16-D102Y, S-D614G, N-S194L	G (B.1.558)
USA/CA-LAC-USC25	6/22/20	28.59	28.63	NSP2-A360V, **NSP3-D1148N**, NSP6-L37F, NSP12-P323L, N-R203K, N-G204R, S-L5F, S-D614G	GR (B.1.1)

Novel amino acid mutations that were not observed among 28,176 global SARS-CoV-2 sequences in GISAID are in bold. C_t_-1 targeted the ORF1/a-b non-structural region and C_t_-2 a conserved region in the structural protein envelope E-gene. qRT-PCR was performed using the Roche COBAS system.

We observed two putative deletions in one sequence (USA/CA-LAC-USC2) that potentially originated from homopolymer sequencing errors. However, these errors were rare as we observed only two deletions out of 754,702 base calls (2.65 $\times$ 10^–6^ per base). We have thus corrected these putative errors.

### COVID-19 specimen profiling

We produced whole genome sequences of 25 Los Angeles County COVID-19 patients who tested positive at Keck Medicine of USC Clinical Laboratories by accessing their remnant NP/OP swab specimens. This study (HS-20-00326) was approved by the Institutional Review Board of the University of Southern California. Table 1 presented each specimen’s collection date from April to June 2020 and COVID-19 test cycle threshold (C_t_) values.

**Figure 2:**
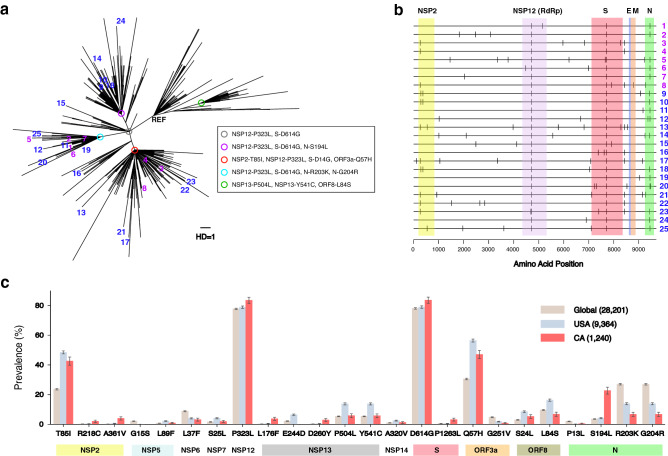
Maximum likelihood tree analysis and amino acid mutations of 25 SARS-CoV-2 whole genome sequences obtained by CorvGenSurv. **(a)** Maximum likelihood tree of 25 SARS-CoV-2 sequences obtained by CorvGenSurv along with sequences collected in California, US. A total of 1215 SARS-CoV-2 sequences collected from California, USA were downloaded from GISAID^20,21^ as of July 27th, 2020. Our sequences were obtained from 25 remnant specimens from COVID-19 testing between April 13th and June 22nd, 2020 from Los Angeles County, California, USA. Specimens collected from April to May 2020 were colored purple and those collected in June were colored blue. Sequences of specimens USA/CA-LAC-USC1 to USA-CA-LAC-USC25 in Table 1 were denoted by 1 to 25 in this tree. All 25 sequences were classified as G clade with mutations P323L in NSP12 (RdRP) and D614G in S protein (grey circle). Different ancestral sequences were presented by circles in different colors with common mutations of each lineage presented in the box. The unit branch length (one nucleotide base substitution) was denoted as “HD = 1”. (**b)** Each of our 25 sequences’ amino acid mutations from Wuhan-Hu-1 (MN908947) were marked using Highlighter (https://www.hiv.lanl.gov/content/sequence/HIGHLIGHT/highlighter_top.html). The regions of NSP2, NSP12 (RdRP), S, E, M, and N were presented by colored boxes. (**c)** The prevalence of each amino acid mutation with greater than 2% frequency either globally, in the USA, or in California. A total of 28,176 global sequences were downloaded from GISAID^20,21^.

Figure 2a presented the maximum likelihood tree of these 25 Los Angeles sequences along with 1,215 sequences from the state of California archived in GISAID^20,21^ as of July 27th, 2020. The most recent common ancestor sequence of the 25 strains (grey circle in Fig. 2a) had three nucleotide substitutions from the reference sequence, Wuhan-Hu-1, and two amino acid changes, P323L in the RNA-dependent RNA polymerase (RdRP, NSP12) and D614G in the S protein^12,35^. As expected, sequences of specimens collected from April to May (purple numbers in Fig. 2a) were located closer to the root of tree than those of specimens collected in June (blue numbers in Fig. 2a). Ten of the 25 sequences shared additional mutations of R203K and G204R in the N (nucleocapsid) protein (skyblue circle in Fig. 2a) as part of the GR-clade^20,21^. Eight other sequences had mutations of T85I in the NSP2 protein and Q57H in the ORF3a protein (red circle in Fig. 2a) as part of the GH-clade^20,21^. Five other sequences shared the mutation S194L in the N protein (purple circle in Fig. 2a) as part of the G-clade^20,21^. One sequence (USA/CA-LAC-USC15) had a unique mutation lineage amongst the sampled 25 sequences as part of the G-clade^20,21^ (Fig. 2a). Another sequence (USA/CA-LAC-USC16) had a unique mutation lineage as part of the GH clade^20,21^. These 25 sequences reflect the high viral diversity observed in California within the G, GR, and GH clades^35,36^.

Figure 2b marked the amino acid changes of each of our 25 sequences in reference to the Wuhan-Hu-1 sequence. We reported a total of nine new amino acid mutations that were not observed among 28,176 global SARS-CoV-2 sequences in GISAID^20,21^. These include M755I in RdRP (NSP12) and V117F in the ORF8 protein (Table 1).

We compared the prevalence of each amino acid mutation from Wuhan-Hu-1 that had greater than 2% frequency either globally, in the USA, or in California as of July, 2020 (Fig. 2c). Mass circulation of G clade strains were confirmed by around 80% prevalence of P323L in RdRP and D614G in the S protein globally. In USA and California, the prevalence of T85I in NSP2 and Q57H in ORF3a were observed to be above 40%, as shown in Fig. 2c. The prevalence of P1263L mutation in the S protein was sixfold greater in California than the global prevalence. Additionally, S194L mutation in the N protein was much more prevalent in California, compared to other regions.

### CorvGenSurv’s supply cost

The development of a cost-effective SARS-CoV-2 genotyping protocol is crucial to expanding COVID-19 surveillance efforts. The per-specimen supply cost of our SARS-CoV-2 whole genome sequencing method was estimated to be $33.8. This includes RNA extraction from NP/OP swab media ($4.58), long-range RT-PCR ($25.8), index PCR ($2.6), and long-read high-throughput sequencing ($0.82).

We estimated the per-specimen high-throughput sequencing cost by assessing the maximum number of specimens ($${N}_{m}$$) we can process in a single sequencing run. We observed that 31 or more reads would be required to produce an accurate consensus sequence (Fig. 1b). Therefore, we estimated how many reads are required on average to obtain a minimum of 31 reads per consensus sequence. The expected minimum value of $${N}_{g}$$ Poisson random variables is given by $$\sum_{k=1}^{\infty}{[\gamma\,\,\left(k,\lambda\right)/{\Gamma}\left(k\right)]}^{{N}_{g}}$$, where $$\lambda$$ is the mean of the Poisson distribution, $$\gamma \left(k,\lambda \right)$$ is the lower incomplete gamma function, and $$\mathrm{\Gamma }\left(k\right)$$ is the gamma function. Since we need three segments per specimen, $${N}_{g}$$ is given by 3 $$\times\,{N}_{m}$$. We obtained around 1.58 million circular consensus sequence (CCS) reads with 99% accuracy for a library size of ~ 10 kb from a single sequencing run. We estimated that 8779 specimens can be processed in a single sequencing run, where 60 reads per consensus sequence can be obtained on average to recover a minimum of 31 reads per consensus sequence. Similarly, if we aim to recover a minimum of 100 reads per consensus sequence as a conservative approach, around 3657 specimens can be processed in a single sequencing run. Assuming a sequencing cost of $3000, this yields a per specimen sequencing cost of $0.82 for large-scale sequencing efforts.

This estimated low-sequencing cost suggests a cost advantage over current short-read based SARS-CoV-2 whole genome sequencing methods^8,10–12,26–30^. The required time for CorvGenSurv is around 15 h for sample library preparation and 30 h for sequencing. Our cost-effective workflow may therefore enhance the implementation of real-time massive genomic SARS-CoV-2 surveillance.

### SARS-CoV-2 evolution rate

We estimated the rate of SARS-CoV-2 evolution from the 25 genomes we obtained using a Bayesian phylogenetic reconstruction method^37,38^. The SARS-CoV-2 nucleotide evolution rate was estimated to be 7.51 $\times$ 10^–4^ substitutions per site per year [95% highest posterior density (HPD): 2.04 $\times$ 10^–4^ to 1.34 $\times$ 10^–3^]. The median divergence time was estimated to be December 5th, 2019 [95% HPD: December 16th, 2018–March 19th, 2020]. This estimate was 26 days prior to the first SARS-CoV-2 sequence Wuhan-Hu-1’s sample collection time, December 31st, 2019.

**Figure 3:**
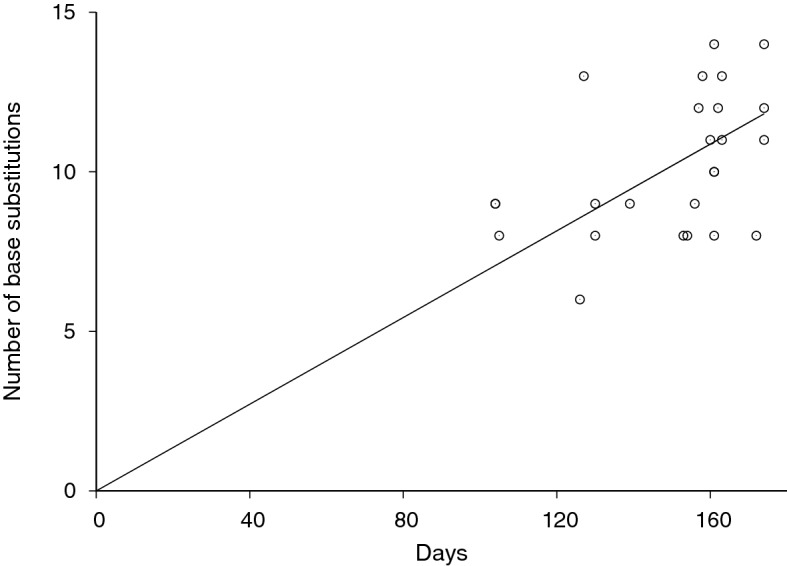
SARS-CoV-2 divergence. Our 25 Los Angeles sequences’ number of base substitutions from the reference sequence Wuhan-Hu-1 (MN908947) was plotted against the collection time of each sequence as days from the reference sequence collection time, December 31st, 2019. The SARS-CoV-2 evolution rate was estimated to be 8.62 $\times$ 10^–4^ substitutions per site per year (95% confidence interval: 7.96 $\times$ 10^–4^ to 9.24 $\times$ 10^–4^) by linear regression (solid line).

The rate of SARS-CoV-2 evolution was also estimated by measuring the rate of divergence from the reference Wuhan-Hu-1 sequence. We plotted our sequences’ number of nucleotide base differences from the Wuhan-Hu-1 sequence against sample collection time difference in Fig. 3. The nucleotide substitution rate in reference to Wuhan-Hu-1 was measured to be 8.62 $\times$ 10^–4^ substitutions per site per year (95% confidence interval: 7.96 $\times$ 10^–4^ to 9.24 $\times$ 10^–4^). This rate was consistent with the above Bayesian phylogenetic estimate.

### Influenza vaccination and evolution

**Figure 4:**
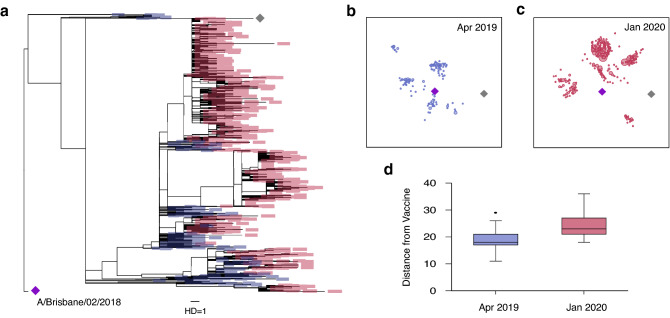
Influenza A (H1N1) evolution and vaccination. **(a)** Maximum likelihood tree of 255 H1N1 Hemagglutinin (HA) sequences sampled in April 2019 (blue boxes), 1140 H1N1 HA sequences sampled in January 2020 (red boxes), 2019–2020 H1N1 Northern hemisphere vaccine strain (A/Brisbane/02/2018, purple diamond) and 2018–2019 vaccine strain (A/Michigan/45/201, grey diamond). All HA nucleotide sequences were downloaded from GISAID^20,21^. The H1N1 HA sequences in January 2020 showed greater tree distances from the 2019–2020 H1N1 vaccine strain, compared to those in April 2019 (**b)** Two-dimensional map of 255 sequences collected in April 2019 along with the 2019–2020 H1N1 vaccine strain’s HA sequence (purple diamond) and 2018–2020 HIN1 vaccine’s HA sequence (grey diamond). The nucleotide distance among all pairs of sequences was scaled to the Euclidean distance by multidimensional scaling. (**c)** Two-dimensional map of 1140 HA sequences collected in January 2020 along with the two vaccine sequences. (**d)** The HA sequences in January 2020 showed greater nucleotide distances from the 2019–2020 vaccine strain than those in April 2019 (p < 0.001, Wilcoxon rank sum test).

It has been reported that several SARS-CoV-2 variants of concern can potentially escape from vaccine-induced immunity^16,17^. The emergence and circulation of such variants has prompted efforts to continuously monitor SARS-CoV-2 evolution via real-time genomic surveillance^39,40^. To further demonstrate its importance, we analyzed influenza evolution in response to vaccination. Influenza has also been shown to escape from vaccine-induced immunity, as confirmed by serologic testing^41^. The WHO changed the 2019–2020 H1N1 Northern Hemisphere Flu vaccine strain from A/Michigan/45/2015 (grey diamond in Fig. 4a–c) to A/Brisbane/02/2018 (purple diamond in Fig. 4a–c)^42^. Our maximum likelihood tree analysis showed that influenza H1NI Hemagglutinin (HA) sequences evolved away from this new vaccine strain. As shown in Fig. 4a, a total of 255 H1N1 Hemagglutinin (HA) sequences sampled in April 2019 were relatively close to the 2019–2020 H1N1 vaccine strain (purple diamond in Fig. 4a). However, we found that HA sequences collected in January 2020 (1140 sequences total) had evolved away from this vaccine strain. This rapid evolution was visualized in a two-dimensional map obtained by multidimensional scaling of the pairwise HA sequence distance matrix (Fig. 4b,c). As plotted in Fig. 4d, the nucleotide base difference from the vaccine sequence was significantly higher among the January 2020 sequences, compared to the April 2019 sequences (median distance 18 vs. 23, p < 0.001).

This observed evolution away from the vaccine strain may explain the 2019–2020 seasonal influenza vaccine’s low effectiveness against influenza A (H1N1). Influenza vaccine efficacy was estimated to be 37% against H1N1 strains during the 2019–20 flu season^43^. Viral evolution in response to vaccination remains a challenge for vaccine design, and the 2019–20 flu season highlights the need for real-time surveillance of viral sequences. Like influenza, SARS-CoV-2 has the potential to evolve in response to vaccination^44^, and thus SARS-CoV-2 evolution must be continuously monitored in real time to prevent vaccine failure and guide future vaccine strain selection.

## Discussion

We developed CorvGenSurv (Coronavirus Genomic Surveillance), a streamlined, high-resolution, and cost-effective SARS-CoV-2 whole genome sequencing method in which viral RNA is directly amplified, indexed, and sequenced. CorvGenSurv’s precision and accuracy have been validated by Sanger sequencing and control genomic RNA sequencing, respectively. Our targeted amplification of the whole genome in three segments minimized the risk of genome coverage dropouts. Additionally, our streamlined long-read sequencing protocol significantly reduced workflow complexity and thus has a potential cost advantage over currently available short-read sequencing approaches^8,10–12,26–28,31^.

We obtained 25 whole genome sequences from NP/OP remnant COVID-19 positive test specimens that were collected from April to June 2020 at Los Angeles County, California, USA. The Los Angeles County pandemic accounted for 33% of all COVID-19 cases in California with around 3500 new cases per day in July 2020^45,46^. Our maximum likelihood tree analysis showed that the 25 strains were highly diverse within the G, GR and GH clades^35^. The G clade and tis lineage have been dominant since late March, 2020 and its D614G mutation in the spike protein has been associated with increased transmissibility^14^. In addition to the G clade’s other common mutation, NSP12-P323L, frequent mutations we observed include N-S194L, N-R203K, N-G204R, NSP2-T85I, and ORF3a-Q57H. From this wide spectrum of viral mutations, we estimated SARS-CoV-2 evolutionary rate by a Bayesian phylogenetic reconstruction method^37,38^ and divergence measures. These two estimates were 7.51 $\times$ 10^–4^ and 8.62 $\times$ 10^–4^ substitutions per site per year respectively, which were consistent with recent reports on the SARS-CoV-2 evolutionary rate^47–49^.

We identified new amino acid mutations in the NSP3, NSP4, RdRP (NSP12), ORF8, and N proteins. Mutations in these proteins have the potential to trigger immune escape, alter viral replication capacity, or modulate viral immune suppression. Numerous T cell epitopes have been annotated in these proteins and thus mutations can lead to viral escape from cellular immune responses^50,51^. While mutations in the N, NSP3, NSP4 or NSP12 proteins can alter viral replication capacity^13,52–55^, those in the ORF8 or N proteins may dysregulate host innate immune responses via type I interferon signal modulation^54^. It is therefore of great importance to monitor mutations from a diverse array of proteins by whole genome sequencing to pinpoint those that are relevant to viral pathogenicity and immune escape.

SARS-CoV-2’s S protein is the key target for many of the vaccines currently in development^18^ and thus mutations in this region are being closely monitored for their potential to render these vaccines ineffective. The observed influenza HA sequence evolution away from this year’s vaccine strain clearly indicates the necessity of screening mutations in viral surface proteins. Genomic surveillance data can also inform SARS-CoV-2 drug resistance. Mutations in RdRP (NSP12) can potentially result in decreased binding affinity with nucleoside analogs such as Remdesivir and Favipiravir^56^. Therefore, genomic surveillance on circulating pandemic strains is crucial for guiding strategies for COVID-19 vaccination and therapeutic intervention and our simple and cost-efficient pipeline has the potential to advance real-time COVID-19 surveillance.

CorvGenSurv can accurately survey new mutations in real-time and thus provide crucial data for disease transmission, new outbreak detection, and vaccine candidate selection. As an alternative to the more common short-read sequencing approaches^8,10–12,26–30^, CorvGenSurv can facilitate the widespread adoption of real-time COVID-19 genomic surveillance and can therefore serve as an important tool for public health decision-making.

## Methods

### COVID-19 specimens at Keck School of Medicine Hospital of USC

We accessed 25 de-identified nasopharyngeal (NP) and oropharyngeal (OP) remnant specimens which tested positive for COVID-19 via the Roche cobas^®^ SARS-CoV-2 qualitative EUA assay at USC Clinical Laboratories, Keck Medicine of USC. These specimens were collected from Los Angeles County, California, USA between April 13th and June 22nd, 2020 (Table 1). This study (HS-20-00326) was approved by the Institutional Review Board of the University of Southern California as non-human subjects research. Table 1 lists each specimen’s collection date and cycle threshold (C_t_) values for the ORF1 a/b non-structural SARS-CoV-2 unique region (C_t_-1) and the pan-Sarbecovirus conserved region in the structural protein envelope E-gene (C_t_-2).

### SARS-CoV-2 global strains

We downloaded near full-length SARS-CoV-2 sequences from GISAID that were registered by July 27th, 2020. The sequences were globally aligned using MUSCLE^57^, and their 5’- and 3’-ends trimmed-out, yielding a 597–29340 segment of the Wuhan-Hu-1 reference strain. We removed any sequences that were shorter than this region and sequences with one or more ambiguous bases to obtain a total of 28,176 near full-length SARS-CoV-2 sequences, including 9339 originating from the USA and 1215 from California. In Table 1, all amino acid mutations from the reference sequence were annotated and novel mutations in our 25 sequences were reported.

### SARS-CoV-2 whole genome sequencing

SARS-CoV-2 RNA was extracted from remnant NP/OP swab specimens in universal transport media using a MagMAX™ Viral/Pathogen Nucleic Acid Isolation Kit with a KingFisher Duo Prime automated nucleic acid purification system (Thermo Fisher Scientific). A total of 20 μl (15 $\le$ C_t_ $<$ 20), 100 μl (20 $\le$ C_t_ $<$ 25), or 200 μl (C_t_ ≥ 25) of viral media was diluted in 1× PBS buffer to a total volume of 400 μl and added to a deep-well plate for extraction using the manufacturer’s protocol. Around 80 μl of extracted SARS-CoV-2 RNA was recovered. This RNA was used as input for three separate 10 kb long RT-PCRs using the SuperScript™ IV One-Step RT-PCR System (Thermo Fisher Scientific). As listed in Supplementary Table S1, the RT-PCR primers were 1_LEFT/33_RIGHT or For-c/Rev-c for the first segment, For-d/67_RIGHT or 33_LEFT/67_RIGHT for the second segment, and 67_LEFT/Rev-e or 67_LEFT/98_RIGHT for the third segment. Here, 1_LEFT, 33_RIGHT, 33_LEFT, 67_RIGHT, 67_LEFT, and 98_RIGHT were the ARTIC network primers (V3, https://artic.network/ncov-2019), where the "nCoV-2019_" suffix was removed for simplicity. Primers, For-c, Rev-c, For-d, and Rev-e were designed in-house. These primers span the near full-length SARS-CoV-2 genome in three segments with overlap for genome assembly. The master mix was composed of 25 μl RT-PCR master mix, 2.5 μl each of 10 μM forward and reverse primers, 0.5 μl SuperScript IV RT mix, and 19.5 μl of viral RNA to a total volume of 50 μl. The samples were cycled according to the manufacturer’s instructions: 50 °C for 10 min of RT, followed by 98 °C for 2 min for RT inactivation, then 35 cycles of 98 °C for 10 s, 55 °C for 10 s, 72 °C for 5 min, then a final extension of 72 °C for 5 min, held at 4 °C until the next step.

The RT-PCR products were then purified and concentrated with Ampure XP beads (Beckman Coulter) with a 0.8 × bead volume, two 70% ethanol washes, and elution in 25 μl Nuclease-Free H_2_O. These purified RT-PCR products were subjected to long-range index PCR using PrimeSTAR GXL DNA Polymerase (Takara Bio) according to the manufacturer’s instructions. After index PCR, product bands were confirmed via 1% agarose gel electrophoresis with the E-Gel Electrophoresis System (ThermoFisher, CA). Samples were equimolar pooled and shipped to DNA Technologies and Expression Analysis Core at UC Davis Genome Center for long-read, single-molecule, real-time (SMRT) sequencing on the PacBio Sequel II system with a 30-h movie.

### SARS-CoV-2 sanger sequencing

Specimen, USA/CA-LAC-USC1, was selected for near full-length SARS-CoV-2 sequence confirmation via Sanger sequencing. Purified RT-PCR product for each of the three segments of USA/CA-LAC-USC1 was amplified via long-range PCR with the master mix composed of 10 μl of PrimeSTAR GXL buffer, 4 μl of dNTPs, 2.5 μl each of 2 μM forward and reverse primers, 1 μl of PrimeSTAR GXL DNA polymerase, 26 μl nuclease free H_2_O, and 4.0 μl of purified RT-PCR product to a total volume of 50 μl. The samples PCR cycled with 25 cycles of 98 °C for 10 s and 68 °C for 10 min, held at 4 °C until the next step. These products were Ampure cleaned with a 0.8 × bead volume and diluted to 20 ng/μl. A total of 10 μl of the diluted product was combined with 5 μl of each Sanger sequencing primer in 5 μM. As listed in Supplementary Table S2, a total of 49 primers were selected from the V3 ARTIC network primers (https://artic.network/ncov-2019) with minor modifications to minimize self-dimer and hairpin formation. The samples were then shipped to GENEWIZ (South Plainfield, NJ) for Sanger sequencing. The obtained sequences were subject to contig assembly using the CAP3 Sequence Assembly^58^ followed by manual inspection, yielding USA/CA-LAC-USC1’s near full-length SARS-CoV-2 genome (55–29,836).

### Consensus sequence building

Each SARS-CoV-2 genome segment was de-multiplexed based on their indexes, creating one fasta file for each of the three segments per specimen. 100 reads were randomly picked from each fasta file and aligned using MUSCLE^57^ to obtain a consensus sequence for each segment. The three segment consensus sequences were then assembled to produce each specimen’s near full-length SARS-CoV-2 whole genome sequence (405–29,431).

### Maximum likelihood tree analysis and Bayesian phylogenetic analysis

We used the PHYML program to produce maximum likelihood trees^59^. The general time-reversible model was used with the ‘ML’ option, and invariable sites were estimated with 12 substitution rate categories. The tree was generated by the BIONJ option. We used FigTree to present the obtained tree file.

BEAST42 v.1.10.4. was used to estimate the rate of SARS-CoV-2 evolution and divergence time^37^. The 25 SARS-CoV-2 near full-length genome sequences obtained in this study were input to BEAST along with its sample collection time (Table 1). Uncorrelated relaxed clock model with log-normal distribution was assumed with flexible skygrid coalescent tree priors and a single GTR + Γ substitution model^38^. The Monte Carlo (MCMC) length of chain was 2 $\times$ 10^6^. The median evolution rate and divergence time along with respective 95% highest posterior density (HPD) were reported.

### Multi-dimensional scaling

We globally aligned 5772 complete HA genome sequences that were collected between April 2019 and March 2020 along with two H1N1 vaccine strains A/Michigan/45/2015 and A/Brisbane/02/2018. These sequences were downloaded from GISIAD^20,21^. The pairwise nucleotide base differences, Hamming Distance (HD), was measured and this HD matrix was input to a multi-dimensional scaling (MDS) algorithm from the python scikit-learn package (0.19.1). This MDS algorithm converted the HD matrix into a two-dimensional representation of the HA sequences by minimizing stress, the sum of squared distance of the disparities. Each sequence’s position was updated until the stress difference was less than 0.1, and by repeating this procedure 400 times, the configuration with the minimum stress value was selected.

## Supplementary Information

Supplementary Tables.

## Data Availability

SARS-CoV-2 whole genome sequences sequenced in this study are available in GISAID (accession numbers EPI_ISL_569664-EIP_ISL_569688).
