# Supplementary material for: High-precision and cost-efficient sequencing for real-time COVID-19 surveillance
Source: Sci Rep. 2021 Jul 1;11:13669. doi: 10.1038/s41598-021-93145-4 (PMC8249533; doi:10.1038/s41598-021-93145-4)
Supplement: Supplementary file 1 — Supplementary Tables. [file 41598_2021_93145_MOESM1_ESM.docx]

**Supplementary Information**

**High-precision and cost-efficient sequencing for real-time COVID-19 surveillance**

Sung Yong Park^1#^, Gina Faraci^1#^, Pamela M. Ward^2^, Jane F. Emerson^2^, and Ha Youn Lee^1*^

^1^Department of Molecular Microbiology and Immunology, Keck School of Medicine, University of Southern California, Los Angeles, USA

^2^Department of Clinical Pathology, Keck School of Medicine, University of Southern California, Los Angeles, USA

| Name | Segment | Forward/  Reverse | Position | Sequence |
| --- | --- | --- | --- | --- |
| 1_LEFT | 1 | Forward | 31-54 | 5’-ACCAACCAACTTTCGATCTCTTGT-3’ |
| 33_RIGHT | 1 | Reverse | 10147-10171 | 5’-TGGACAGTAAACTACGTCATCAAGC-3’ |
| For-c | 1 | Forward | 381-404 | 5’-TATCAGAGGCACGTCAACATCTTA-3’ |
| Rev-c | 1 | Reverse | 10244-10267 | 5’-ACCAGCCTGTACCAAGAAATTATG-3’ |
| For-d | 2 | Forward | 9634-9658 | 5’-AGCACATATTCAGTGGATGGTTATG-3’ |
| 33_LEFT | 2 | Forward | 9785-9806 | 5’-ACTTTTGAAGAAGCTGCGCTGT-3’ |
| 67_RIGHT | 2 | Reverse | 20543-20572 | 5’-CAACCTTAGAAACTACAGATAAATCTTGGG-3’ |
| 67_LEFT | 3 | Forward | 20173-20200 | 5’-GTTGTCCAACAATTACCTGAAACTTACT-3’ |
| Rev-e | 3 | Reverse | 29432-29458 | 5’-AAGAAGAGTCACAGTTTGCTGTTTCTT-3’ |
| 98_RIGHT | 3 | Reverse | 29837-29866 | 5’-TTCTCCTAAGAAGCTATTAAAATCACATGG-3’ |

**Table S1. Reverse Transcription-PCR (RT-PCR) Primers.** Primer position is relative to the reference genome, Wuhan-Hu-1 (MN908947). Primers, 1_LEFT, 33_RIGHT, 33_LEFT, 67_RIGHT, 67_LEFT, 98_RIGHT were obtained from the ARTIC network (V3, https://artic.network/ncov-2019). Primers, For-c, Rev-c, For-d, and Rev-e were designed in-house.

| Name | Forward / Reverse | Position | Sequence (5’🡪3’) |
| --- | --- | --- | --- |
| 1_LEFT | Forward | 31-54 | ACCAACCAACTTTCGATCTCTTGT |
| 3_LEFT | Forward | 643-664 | CGGTAATAAAGGAGCTGGTGGC |
| 5_LEFT | Forward | 1243-1264 | TGGTGAAACTTCATGGCAGACG |
| 7_LEFT | Forward | 1876-1897 | ATCAGAGGCTGCTCGTGTTGTA |
| 9_LEFT-mod | Forward | 2523-2542 | CAGAGGAAGTTGTCTTGAAA |
| 11_LEFT | Forward | 3145-3166 | GGAATTTGGTGCCACTTCTGCT |
| 13_LEFT-mod | Forward | 3772-3792 | TCGCACAAATGTCTACTTAGC |
| 15_LEFT-mod | Forward | 4300-4321 | GCTTAAAAAGTGTAAAAGTGCC |
| 17_LEFT-mod | Forward | 4943-4966 | CTTTCTTTGAGAGAAGTGAGGACT |
| 19_LEFT-mod | Forward | 5578-5597 | GGGCACACTTTCTTATGAAC |
| 21_LEFT-mod | Forward | 6153-6172 | TAAATGGTGATGTGGTGGCT |
| 23_LEFT-mod | Forward | 6723-6745 | CTACTAACATAGTTACACGGTGT |
| 25_LEFT-mod | Forward | 7309-7331 | ATTGTTTTTCAGCTATTTTGCAG |
| 27_LEFT-mod | Forward | 7944-7967 | ACTACAGTCAGCTTATGTGTCAAC |
| 29_LEFT | Forward | 8596-8619 | ACTTGTGTTCCTTTTTGTTGCTGC |
| 33_RIGHT | Reverse | 10147-10171 | TGGACAGTAAACTACGTCATCAAGC |
| 33_LEFT | Forward | 9785-9806 | ACTTTTGAAGAAGCTGCGCTGT |
| 35_LEFT | Forward | 10363-10384 | TGTTCGCATTCAACCAGGACAG |
| 37_LEFT | Forward | 11000-11022 | ACACACCACTGGTTGTTACTCAC |
| 39_LEFT-mod | Forward | 11562-11584 | GCCCTATTTTCTTCATAACTGGT |
| 41_LEFT-mod | Forward | 12104-12126 | GAGTTTAGTTCCCTTCCATCATA |
| 43_LEFT | Forward | 12711-12732 | TACGACAGATGTCTTGTGCTGC |
| 45_LEFT-mod | Forward | 13320-13343 | TACCTACAACTTGTGCTAATGACC |
| 47_LEFT-mod | Forward | 13923-13946 | CTGGTATGATTTTGTAGAAAACCC |
| 49_LEFT | Forward | 14546-14570 | AGGAATTACTTGTGTATGCTGCTGA |
| 51_LEFT | Forward | 15172-15193 | TCAATAGCCGCCACTAGAGGAG |
| 53_LEFT | Forward | 15828-15851 | AGCAAAATGTTGGACTGAGACTGA |
| 55_LEFT-mod | Forward | 16420-16443 | CAACTTTACTTAGGAGGTATGAGC |
| 57_LEFT | Forward | 17066-17087 | ATTCTACACTCCAGGGACCACC |
| 59_LEFT-mod | Forward | 17675-17696 | TCACGCATGATGTTTCATCTGC |
| 61_LEFT-mod | Forward | 18266-18286 | GCGAAGAAGCTATAAGACATG |
| 63_LEFT | Forward | 18897-18918 | TGTTAAGCGTGTTGACTGGACT |
| 67_RIGHT | Reverse | 20543-20572 | CAACCTTAGAAACTACAGATAAATCTTGGG |
| 67_LEFT | Forward | 20173-20200 | GTTGTCCAACAATTACCTGAAACTTACT |
| 69_LEFT-mod | Forward | 20787-20810 | TGTCGCAAAATATACTCAACTGTG |
| 71_LEFT-mod | Forward | 21363-21386 | TCCAATTCAGTTGTCTTCCTATTC |
| 73_LEFT-mod | Forward | 21969-21991 | GTAATGATCCATTTTTGGGTGTT |
| 75_LEFT-mod | Forward | 22517-22541 | AGAGTCCAACCAACAGAATCTATTG |
| 77_LEFT | Forward | 23123-23144 | CCAGCAACTGTTTGTGGACCTA |
| 79_LEFT-mod | Forward | 23784-23804 | ACATTTGTGGTGATTCAACTG |
| 81_LEFT-mod | Forward | 24396-24416 | TTGGAAAACTTCAAGATGTGG |
| 83_LEFT-mod | Forward | 24963-24985 | ACAACACAGTTTATGATCCTTTG |
| 85_LEFT-mod | Forward | 25602-25621 | ACTAGCACTCTCCAAGGGTG |
| 87_LEFT | Forward | 26198-26219 | CGACTACTAGCGTGCCTTTGTA |
| 89_LEFT-mod | Forward | 26840-26861 | GCGTTCCATGTGGTCATTCAAT |
| 91_LEFT-mod | Forward | 27449-27469 | ACTACCAAGAGTGTGTTAGAG |
| 93_LEFT | Forward | 28085-28103 | GGCTGGTTCTAAATCACCC |
| 95_LEFT | Forward | 28678-28699 | TGAGGGAGCCTTGAATACACCA |
| 98_RIGHT | Reverse | 29837-29866 | TTCTCCTAAGAAGCTATTAAAATCACATGG |

**Table S2.** **SARS-CoV-2 Sanger sequencing primers.** Primers from the ARTIC network V3 primer list. A portion of primers were modified to optimize the hairpin score, self-dimer score, and melting temperature (marked as “-mod”).
